# Alzheimer Disease Blood Biomarkers and Cognition Among Individuals With Diabetes and Overweight or Obesity

**DOI:** 10.1001/jamanetworkopen.2024.58149

**Published:** 2025-02-06

**Authors:** Michelle M. Mielke, Joni K. Evans, Rebecca H. Neiberg, Doris P. Molina-Henry, Santica M. Marcovina, Karen C. Johnson, Owen T. Carmichael, Stephen R. Rapp, Bonnie C. Sachs, Jingzhong Ding, Heather M. Shappell, Jose A. Luchsinger, Mark A. Espeland, Kathleen M. Hayden

**Affiliations:** 1 Department of Epidemiology and Prevention, Wake Forest University School of Medicine, Winston-Salem, North Carolina; 2 Department of Biostatistics and Data Science, Wake Forest University School of Medicine, Winston-Salem, North Carolina; 3 Winston-Salem State University, Winston-Salem, North Carolina; 4 Alzheimer’s Therapeutic Research Institute, Keck School of Medicine, University of Southern California, Los Angeles; 5 Medpace Reference Laboratories, Cincinnati, Ohio; 6 Department of Preventive Medicine, University of Tennessee Health Science Center, Memphis; 7 Biomedical Imaging Center, Pennington Biomedical Research Center, Baton Rouge, Louisiana; 8 Department of Social Sciences and Health Policy, Wake Forest University School of Medicine, Winston-Salem, North Carolina; 9 Department of Psychiatry and Behavioral Medicine, Wake Forest University School of Medicine, Winston-Salem, North Carolina; 10 Department of Neurology, Wake Forest University School of Medicine, Winston-Salem, North Carolina; 11 Department of Internal Medicine, Division of Gerontology and Geriatric Medicine Research, Wake Forest University School of Medicine, Winston-Salem, North Carolina; 12 Departments of Medicine and Epidemiology, Columbia University Irving Medical Center, New York, New York

## Abstract

**Importance:**

Blood-based biomarkers (BBMs) are clinically available to aid in the diagnosis of Alzheimer disease (AD) and AD-related dementias (ADRD), but their association with cognition among older adults with specific chronic conditions has not been examined.

**Objective:**

To longitudinally examine associations between baseline AD and ADRD BBMs and change in BBMs with cognition among participants with type 2 diabetes (T2D) and overweight or obesity.

**Design, Setting, and Participants:**

The Look AHEAD (Action for Health in Diabetes) study was a clinical trial of older adults with T2D and overweight or obesity randomized to a 10-year intensive lifestyle intervention for weight loss or a diabetes support and education condition. Participants were recruited and followed up at 16 clinical sites across the US. Enrollment occurred from January 1, 2001, to December 31, 2004. The primary intervention spanned the first 4 years after participants’ enrollment (January 1, 2008, to December 31, 2011). The clinical trial was stopped in September 2012 and was converted to an observational study. Blood samples were drawn at baseline and 8 to 12 years later. Cognitive assessments were performed from January 1, 2013, to December 31, 2014, and from January 1, 2018, to December 31, 2020. Data for the present cohort study were analyzed between January and August 2024.

**Exposures:**

Baseline and 8- to 12-year change in plasma levels of amyloid-β (Aβ)_40_, Aβ_42_, Aβ_42/40_ ratio, phosphorylated tau 181 (pTau-181), glial fibrillary acidic protein (GFAP), and neurofilament light chain (NfL).

**Main Outcomes and Measures:**

Cognitive composite *z* score and adjudicated mild cognitive impairment or probable dementia.

**Results:**

The mean (SD) baseline age of 758 participants was 61.5 (6.1) years, and 424 participants [55.9%] were female. Mean (SD) body mass index was 34.8 (5.3). Of the participants, 373 were randomized to diabetes support and education and 385 to intensive lifestyle intervention. Increasing baseline BBM levels were not associated with any cognitive composite *z* score. Increasing levels of NfL (β = −0.032 [SE, 0.013]; *P* = .01) and GFAP (β = −0.087 [SE, 0.025]; *P* < .001), but not the Aβ_42/40_ ratio (β = 0.006 [SE, 0.040]; *P* = .88) or pTau-181 (β = 0.026 [SE, 0.025]; *P* = .31), were associated with worsening cognitive function and incident mild cognitive impairment or probable dementia. The intervention had no association with 8- to 12-year change in BBM levels.

**Conclusions and Relevance:**

In this study of participants with T2D and overweight or obesity, increasing plasma NfL and GFAP levels over time, but not Aβ_42/40_ or pTau-181 levels, were associated with cognitive decline and incident cognitive impairment. These results suggest that plasma NfL and GFAP may be important biomarkers of cognitive change among this patient population.

## Key Points

**Question:**

Are plasma Alzheimer disease (AD) blood-based biomarkers associated with cognitive impairment in individuals with type 2 diabetes and overweight or obesity?

**Findings:**

In this cohort study of 758 individuals with type 2 diabetes and overweight or obesity, baseline blood-based biomarker levels were not associated with a cognitive composite *z* score or adjudicated cognitive impairment. Increasing neurofilament light chain and glial fibrillary acidic protein levels, but not change in the amyloid-β_42/40_ ratio or phosphorylated tau 181, were associated with worsening cognitive function and incident cognitive impairment.

**Meaning:**

Increasing plasma neurofilament light chain and glial fibrillary acidic protein levels are biomarkers of incident cognitive impairment among participants with type 2 diabetes and overweight or obesity.

## Teaser

This cohort study of individuals with type 2 diabetes and overweight or obesity examined whether baseline Alzheimer disease blood-based biomarkers were associated with cognitive decline and impairment.

## Introduction

Biomarkers of Alzheimer disease (AD) and Alzheimer disease–related dementias (ADRD) are important for a more accurate diagnosis of AD and ADRD.^1^ Compared with a lumbar puncture to measure cerebrospinal fluid amyloid-β (Aβ) and phosphorylated tau (pTau) or amyloid positron emission tomography, obtaining blood-based markers (BBMs) is less invasive, less costly, more accessible, and more feasible for serial assessments.^2,3^ Several studies have shown that the plasma Aβ_42/40_ ratio, pTau-181, and phosphorylated Tau 217 (pTau-217) are associated with brain amyloid pathology measured via positron emission tomography or cerebrospinal fluid^4,5,6,7,8,9,10^ and risk of cognitive decline and incident dementia.^4^ In addition, nonspecific biomarkers of neurodegeneration (ie, neurofilament light chain [NfL]) and neuroinflammation (ie, glial fibrillary acidic protein [GFAP]) have also been associated with cognitive decline.^11,12,13^ Although these BBMs are now clinically available as laboratory-developed tests to aid in the diagnosis of AD and ADRD, most BBM research studies have included relatively healthy participants to date. Older adults with multiple chronic conditions are most at risk for AD and ADRD, but few studies have assessed the associations of BBMs with cognition among individuals with multiple chronic conditions.^14^

Diabetes and obesity have 2 of the highest dementia population attributable fractions, especially for Black and Hispanic individuals.^15^ We previously examined associations between AD BBM levels and demographic and clinical characteristics in the Look AHEAD (Action for Health in Diabetes) study, an observational continuation of a clinical trial cohort of older individuals with type 2 diabetes and overweight or obesity.^16^ We examined whether baseline AD BBMs and 8- to 12-year change were associated with cognitive decline and adjudicated cognitive impairment. We assessed whether previously identified factors found to affect the BBM levels in this cohort (eg, chronic kidney disease) modified the associations between the BBMs and outcomes. Additionally, because the cohort comprised members of a clinical trial, we examined the legacy association of the intensive lifestyle intervention with change in BBM levels.

## Methods

### Study Design

Look AHEAD was a randomized clinical trial of a lifestyle intervention for participants with type 2 diabetes and overweight or obesity.^17^ The Look AHEAD design, methods,^18^ and CONSORT diagram^19^ have been previously published. Briefly, the trial was designed to determine the appropriateness of intentional weight loss among older adults with type 2 diabetes and overweight or obesity. Fatal and nonfatal cardiovascular events were the primary trial end points. Participants were required to have body mass index (BMI; calculated as weight in kilograms divided by height in meters squared) of 25 or greater (≥27 if receiving insulin), glycated hemoglobin of 11% or less (to convert to proportion of total hemoglobin, multiply by 0.01), systolic/diastolic blood pressure less than 160/100 mm Hg, and triglyceride levels less than 600 mg/dL (to convert to millimoles per liter, multiply by 0.0113) to be eligible to participate. Participants also had to demonstrate, over a 2-week run-in period, that they could make a daily record of their diet and physical activity. Each prospective participant met with a behavioral psychologist or interventionist to confirm that the intervention requirements were understood and that participants did not have any competing life stressors that would impair adherence to the protocol. Enrollment occurred from January 1, 2001, to December 31, 2004. The primary intervention spanned the first 4 years after participants’ enrollment (January 1, 2008, to December 31, 2011). Thereafter, participants continued meeting with staff, although less frequently. The study was stopped in September 2012 for futility of the primary outcome of major cardiovascular events, and the trial was converted to an observational study (Figure). Local institutional review boards approved the protocols, and all participants provided written informed consent. The Strengthening the Reporting of Observation Studies in Epidemiology (STROBE) reporting guideline was followed.

**Figure.**
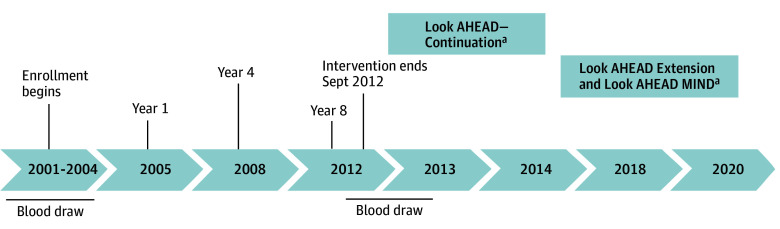
Timeline of the Look AHEAD (Action for Health in Diabetes) Study ^a^Ancillary studies with cognitive assessments, including Look AHEAD–Continuation (n = 3750) as well as Look AHEAD–Extension and Look AHEAD MIND (n = 2451).

### Setting, Participants, and Intervention

A total of 5145 participants aged 45 to 76 years were recruited from 16 clinical sites across the US and were randomized to either an intensive lifestyle intervention (ILI) or a diabetes support and education (DSE) condition (eAppendix in Supplement 1). The ILI was a multidomain intervention that emphasized dietary modification and physical activity with a goal of inducing a mean of 7% or greater weight loss at 1 year and maintenance of their weight loss level throughout the study.^20^ Participants had a daily calorie goal of 1200 to 1800 kcal based on initial weight. Their dietary requirements specified less than 30% total calories from fat (<10% saturated fat) and a minimum of 15% total calories from protein. The physical activity goal was similar in intensity to brisk walking for at least 175 minutes per week. Participants randomized to the DSE condition were invited to attend 3 yearly group sessions that focused on diet, physical activity, and social support.^21^ These sessions had no specific instructions or goals for weight loss, physical activity, or dietary modification.

### Blood-Based Biomarkers

Blood samples were drawn at baseline and proximal to the end of the intervention, approximately 8 to 12 years later. Of participants with cognitive data, samples for blood assays were first prioritized for participants who completed the brain magnetic resonance imaging ancillary study,^18^ followed by random selection.

EDTA plasma samples were sent to Medpace Reference Laboratories. All samples were run for all biomarkers in a single batch using the same lot of reagents. Concentrations of Aβ_42_, Aβ_40_, GFAP, and NfL were measured using an assay kit (Simoa Human Neurology 4-Plex E Advantage Kit) on a bead-based immunoassay analyzer (Quanterix Simoa HD-X, Quanterix Corp). The determination of the concentration of the pTau-181 was performed using an assay kit (Simoa pTau-181 V2 Advantage Kit). Within-run coefficients of variation ranged from 3% to 19%, and between-run coefficients of variation ranged from 6% to 13%.

### Cognitive Assessments

The full Look AHEAD cohort was invited to participate in cognitive assessments from January 1, 2013, to December 31, 2014 (n = 3750), and from January 1, 2018, to December 31, 2020 (n = 2451). Staff were centrally trained and certified for the administration of the standardized cognitive assessments; they also were blinded to participant’s randomization status.^22^

The cognitive battery included the Rey Auditory Verbal Learning Test,^23^ Digit Symbol Coding,^24^ the Modified Stroop Color and Word Test,^25^ and the Trail Making Test Part A and Part B.^26^ Global cognitive function was evaluated with the modified Mini Mental State Examination.^27^ Test scores were standardized as *z* scores using baseline means and SDs. Scores were then averaged to derive a cognitive composite score.^22^

A masked expert panel adjudicated cognitive status to identify cognitive impairment and dementia.^28^ Participants whose modified Mini Mental State Examination scores fell below age- and education-specific cut points underwent review, which was supplemented by telephone administration of the Functional Assessment Questionnaire to a friend or family member to query functional status and instrumental activities of daily living.^29^ Two adjudicators independently reviewed all cognitive tests and Functional Assessment Questionnaire scores, and all relevant data (physical function, medications, depression, and hospitalizations) to make their primary classification (no impairment, all-cause mild cognitive impairment [MCI], or probable dementia), using a successful protocol adapted from another multicenter trial.^30^

Cognitive assessments were conducted at multiple time points. The full Look AHEAD cohort was invited to provide cognitive assessments as part of the Look AHEAD–Continuation (n = 3750) in 2013 to 2014 and Look AHEAD MIND (n = 2451), a cognitive ancillary that took place from 2018 to 2020. Smaller subsets of participants took part in cognitive assessments in 2009 to 2012 (n = 971) and 2011 to 2013 (n = 601) when enrolled in ancillary studies.^28^ Cognitive adjudication of MCI and dementia is based on single assessments obtained in Look AHEAD–Continuation.

### Demographic and Clinical Characteristics

Demographic characteristics collected at baseline were self-reported and included age, sex, race, ethnicity, and educational level. Race was included because of the importance of a diverse sample. *APOE* (OMIM 107741) ε4 carrier status was determined for participants who provided consent using TaqMan genotyping (rs7412 and rs429358).^31^ Baseline weight was measured with digital scales. Diabetes duration and treatment type, hypertension, and hyperlipidemia were self-reported. Baseline report of cardiovascular disease (CVD) included self-report of myocardial infarction, heart bypass surgery, coronary artery bypass graft, carotid endarterectomy, lower leg angioplasty, aortic aneurysm, congestive heart failure, or stroke. Blood specimens were analyzed centrally at the Northwest Lipid Metabolism and Diabetes Research Laboratories at the University of Washington for glycated hemoglobin and estimated glomerular filtration rate (eGFR). eGFR was dichotomized at less than 90 vs 90 or higher due to the small number of participants who had an eGFR less than 60 at baseline.

### Statistical Analysis

Analyses were conducted between January and August 2024. We compared demographic and clinical characteristics of the sample by intervention arm and inclusion status. Continuous variables were evaluated using 2-tailed, unpaired *t* tests, and categorical variables were evaluated with χ^2^ tests. Mixed-effects models with random intercepts were fitted to assess whether baseline BBM levels or change in levels from baseline to years 8 to 12 were associated with cognitive *z* scores using restricted maximum likelihood. We included all cognitive data in our analyses for participants with BBM measures, which ranged from 1 to 4 longitudinal assessments among participants. Inferences and 95% CIs were based on the mean scores over repeat assessments and separately on their slope over time since randomization. Models controlled for randomization arm, age, sex, race, ethnicity, educational level, baseline BMI, diabetes duration, hypertension, CVD history, *APOE* ε4 carrier status, and baseline eGFR. We then evaluated associations between biomarker levels at baseline and change in biomarker levels from baseline to years 8 to 12 (approximately the end of intervention) and our adjudicated cognitive outcome (composite of incident all-cause MCI or probable dementia) for each of our BBMs using logistic regression. Models controlled for randomization arm, age, sex, race, ethnicity, educational level, baseline BMI, diabetes duration, hypertension, CVD history, *APOE* ε4 carrier status, baseline eGFR, and time from baseline blood sample to the assessment that triggered cognitive adjudication. For both models, tests for interactions were used to assess the consistency of associations in strata of eGFR (<90 vs ≥90) given its association with BBM levels. All analyses were performed with SAS, version 9.4 (SAS Institute Inc). A 2-tailed *P* ≤ .05 was considered statistically significant.

## Results

### Participant Characteristics

There were 758 participants (373 DSE and 385 ILI) included in our analyses. Baseline characteristics for the included participants are given in Table 1; eTable 1 in Supplement 1 compares baseline characteristics between the 758 included participants and the 4148 participants without AD or ADRD BBMs. The mean (SD) age was 61.5 (6.1) years; 424 participants (55.9%) were female and 334 (44.1%) were male; 187 of the 735 individuals with reported education (25.4%) had less than 13 years of education; 126 (16.6%) were African American or Black; 4 (0.5%) were American Indian, Native American, or Alaska Native; 6 (0.8%) were Asian or Pacific Islander; 511 (67.4%) were White; 14 (1.8%) were multiracial; and 97 (12.8%) identified as Hispanic. The mean (SD) BMI at baseline was 34.8 (5.3). Of the 752 participants with information on diabetes duration, 427 (56.8%) had diabetes for at least 5 years, and 651 (85.9%) were taking a diabetes medication. More participants in the ILI group reported baseline peripheral neuropathy than among those in the DSE group (74 [19.2%] vs 48 [12.9%]; *P* = .02). There were no significant group differences in baseline BBM levels between the ILI and DSE groups or BBM levels at the end of the intervention (years 8-12). Thus, subsequent analyses of biomarkers were not stratified by intervention arm. At follow-up, 297 individuals were adjudicated as having cognitive impairment (eTable 2 in Supplement 1).

**Table 1.** Baseline Characteristics of 758 Look AHEAD Participants by Randomization Group

Characteristic	No. (%) of participants^a^			*P* value
	Total (N = 758)	DSE (n = 373)	ILI (n = 385)	
Age, mean (SD), y	61.5 (6.1)	61.4 (6.3)	61.6 (6.0)	.64
Sex				
Women	424 (55.9)	212 (56.8)	212 (55.1)	.63
Men	334 (44.1)	161 (43.2)	173 (44.9)	
Race and ethnicity				
African American or Black (not Hispanic)	126 (16.6)	60 (16.1)	66 (17.1)	.69
American Indian, Native American, or Alaska Native	4 (0.5)	2 (0.5)	2 (0.5)	
Asian or Pacific Islander	6 (0.8)	1 (0.3)	5 (1.3)	
Hispanic	97 (12.8)	48 (12.9)	49 (12.7)	
White	511 (67.4)	254 (68.1)	257 (66.8)	
Multiracial	14 (1.8)	8 (2.1)	6 (1.6)	
Length of education, y^b^				
<13	187 (25.4)	81 (22.5)	106 (28.3)	.19
13-16	263 (35.8)	132 (36.7)	131 (34.9)	
>16	285 (38.8)	147 (40.8)	138 (36.8)	
BMI, mean (SD)	34.8 (5.3)	35.0 (5.4)	34.6 (5.3)	.32
Systolic blood pressure, mean (SD), mm Hg	129.0 (17.7)	129.8 (17.7)	128.2 (17.7)	.20
Diastolic blood pressure, mean (SD), mm Hg	69.5 (9.6)	69.4 (9.4)	69.6 (9.9)	.87
Glycated hemoglobin, mean (SD), %^c^	7.2 (1.1)	7.2 (1.2)	7.2 (1.1)	.65
Diabetes duration of ≥5 y^d^	427 (56.8)	212 (57.1)	215 (56.4)	.84
Diabetes treatment^e^				
No medication	101 (13.4)	52 (14.1)	49 (12.8)	.87
Oral medication, no insulin	539 (71.7)	262 (71.0)	277 (72.3)	
Oral medication and insulin	112 (14.9)	55 (14.9)	57 (14.9)	
Dyslipidemia	684 (90.2)	336 (90.1)	348 (90.4)	.89
Hypertension	637 (84.0)	315 (84.5)	322 (83.6)	.76
CVD history	127 (16.8)	61 (16.4)	66 (17.1)	.77
Peripheral neuropathy	122 (16.1)	48 (12.9)	74 (19.2)	.02
*APOE* ε4 carrier status^f^	147 (22.7)	65 (20.9)	82 (24.3)	.30
eGFR <90^g^	316 (45.1)	155 (44.9)	161 (45.2)	.94
Aβ_40_, mean (SD), pg/dL	66.6 (19.4)	66.7 (20.7)	66.5 (18.0)	.87
Aβ_42_, mean (SD), pg/dL	4.9 (1.4)	4.9 (1.4)	4.8 (1.4)	.24
Aβ_42/40_, mean (SD), pg/dL	0.1 (0.0)	0.1 (0.0)	0.1 (0.0)	.80
GFAP, mean (SD), pg/dL	93.6 (40.6)	92.8 (39.9)	94.3 (41.4)	.60
NfL, mean (SD), pg/dL	13.8 (6.4)	14.1 (7.3)	13.4 (5.5)	.11
pTau-181, mean (SD), pg/dL	9.3 (5.1)	9.1 (4.9)	9.4 5.2)	.44

Abbreviations: Aβ, amyloid-β; BMI, body mass index (calculated as weight in kilograms divided by height in meters squared); CVD, cardiovascular disease; DSE, diabetes support and education; eGFR, estimated glomerular filtration rate; GFAP, glial fibrillary acidic protein; ILI, intensive lifestyle intervention; NfL, neurofilament light chain; pTau-181, phosphorylated Tau 181.

^a^ Unless otherwise indicated.

^b^ Information on length education was available for 735 participants (360 in the DSE group and 375 in the ILI group).

^c^ To convert to proportion of total hemoglobin, multiply by 0.01.

^d^ Information on diabetes duration was available for 752 articipants (371 in the DSE group and 381 in the ILI group).

^e^ Information on medication for diabetes was available for 752 participants (369 in the DSE group and 383 in the ILI group).

^f^ Information on *APOE* ε4 carrier status was available for 648 participants (311 in the DSE group and 337 in the ILI group).

^g^ eGFR was available for 701 participants (345 in the DSE group and 356 in the ILI group).

### Association Between BBMs and Cognitive Composite *z* Score

Mixed-effects models for each biomarker’s association with the cognitive composite *z* scores are given in Table 2. None of the baseline AD BBMs were associated with the cognitive composite *z* score 8 to 12 years later. However, each unit increase in plasma NfL (β = −0.032 [SE, 0.013]; *P* = .01) and GFAP (β = −0.087 [SE, 0.025]; *P* < .001) levels during the 8- to 12-year follow-up was associated with poorer cognitive function. There were no associations between Aβ_42_ (β = −0.018 [SE, 0.022]; *P* = .42), Aβ_40_ (β = −0.023 [SE, 0.019]; *P* = .22), Aβ_42/40_ (β = 0.006 [SE, 0.040]; *P* = .88), or pTau-181 (β = 0.026 [SE, 0.025]; *P* = .31) and the cognitive composite *z* score. In additional interaction analyses, none of the associations differed by eGFR strata (<90 vs ≥90).

**Table 2.** Results of Mixed-Effects Models for Each Biomarker Parameter Measured at Baseline and Changes in Biomarker Levels in Association With Performance on a Cognitive Composite^a^

Biomarker	Baseline biomarker levels		Change in biomarker values	
	β coefficient (SE)	*P* value	β coefficient (SE)	*P* value
Aβ_42_	0.024 (0.033)	.48	−0.018 (0.022)	.42
Aβ_40_	−0.023 (0.034)	.50	−0.023 (0.019)	.22
Aβ_42/40_	0.047 (0.032)	.14	0.006 (0.040)	.88
pTau-181	0.033 (0.031)	.29	0.026 (0.025)	.31
NfL	−0.022 (0.035)	.53	−0.032 (0.013)	.01
GFAP	−0.016 (0.034)	.64	−0.087 (0.025)	<.001

Abbreviations: Aβ, amyloid-β; GFAP, glial fibrillary acidic protein; NfL, neurofilament light chain; pTau-181, phosphorylated Tau 181.

^a^ Mixed-effects models for each biomarker are adjusted for randomization arm, age, sex, race, ethnicity, educational level, baseline body mass index, diabetes duration, hypertension, cardiovascular disease history, *APOE* ε4 carrier status, and estimated glomerular filtration rate strata (<90 vs ≥90).

### Association Between BBMs and Adjudicated Cognitive Impairment

Logistic regression models for biomarker levels at baseline and change over time in relation to odds of our adjudicated cognitive impairment outcome (MCI or probable dementia) are reported in Table 3. As above, baseline AD BBMs were not associated with odds of cognitive impairment. However, increasing levels of NfL (odds ratio [OR], 1.10; 95% CI, 1.03-1.18) and GFAP (OR, 1.25; 95% CI, 1.10-1.42) were associated with increased odds of cognitive impairment. There were no associations between baseline Aβ_42,_ Aβ_40,_ Aβ_42/40,_ or pTau-181 and odds of adjudicated cognitive impairment. However, when incorporating additional interaction analyses, we found that the association between increasing plasma pTau-181 and odds of adjudicated cognitive impairment differed by baseline eGFR. Among participants with an eGFR less than 90, the odds of cognitive impairment were 0.75 (95% CI, 0.62-0.93) for each increasing pTau-181 unit, whereas the odds were 1.05 (95% CI, 0.86-1.26) among participants with an eGFR of 90 or greater.

**Table 3.** Results of Logistic Regression Models for Each Biomarker Parameter Measured at Baseline and the End of the Intervention in Association With Odds of Mild Cognitive Impairment or Probable Dementia^a^

Biomarker	Baseline models		Change models	
	Odds ratio (95% CI)	*P* value	Odds ratio (95% CI)	*P* value
Aβ_42_	1.04 (0.87-1.24)	.66	1.08 (0.96-1.20)	.21
Aβ_40_	1.11 (0.93-1.33)	.27	1.09 (0.99-1.20)	.08
Aβ_42/40_	0.91 (0.77-1.07)	.25	1.01 (0.82-1.25)	.93
pTau-181	0.95 (0.81-1.12)	.57	0.90 (0.79-1.03)	.12
NfL	1.16 (0.97-1.38)	.12	1.10 (1.03-1.18)	.008
GFAP	1.15 (0.97-1.38)	.12	1.25 (1.10-1.42)	.001

Abbreviations: Aβ, amyloid-β; GFAP, glial fibrillary acidic protein; NfL, neurofilament light chain; pTau-181, phosphorylated Tau 181.

^a^ Models are adjusted for randomization arm, age, sex, race, ethnicity, educational level, baseline body mass index, diabetes duration, hypertension, cardiovascular history, *APOE* ε4 carrier status, baseline estimated glomerular filtration rate, and time from baseline blood sample to the assessment that triggered cognitive adjudication.

## Discussion

In this cohort of older adults with type 2 diabetes and overweight or obesity, we examined associations of AD and ADRD plasma biomarkers with a cognitive composite *z* score and adjudicated cognitive impairment outcomes. We adjusted for multiple chronic conditions common among older adults and assessed the legacy effect of the intensive lifestyle intervention on biomarker levels. There were several key findings. First, the intensive lifestyle intervention had no legacy association with the AD or ADRD plasma biomarker levels. Second, there were no associations of baseline Aβ_42_, Aβ_40_, Aβ_42/40_, or pTau-181 levels or change in levels with a cognitive composite *z* score or with adjudicated cognitive impairment. Third, increasing levels of plasma NfL and GFAP were associated with decreasing cognitive composite scores and increased odds of our cognitive impairment outcome (MCI or probable dementia). These results suggest that increasing levels of plasma NfL and GFAP among older adults with diabetes and overweight or obesity may be indicative of worsening cognition and therefore are potentially important blood biomarkers.

Although type 2 diabetes and obesity are known risk factors for cognitive decline and dementia, the underlying cause is still not well understood. Previous studies examining type 2 diabetes and AD pathology reported conflicting results. Although 1 study reported that type 2 diabetes was associated with elevated brain amyloid,^32^ other studies reported that type 2 diabetes was more strongly associated with greater cerebrovascular disease, glucose hypometabolism, and brain atrophy than with amyloid pathology.^33,34,35^ In the current study, we also did not find associations between plasma baseline Aβ or pTau-181 levels or change in levels with any cognitive outcomes. These findings concur with a study of older adults with type 1 diabetes that did not find associations between plasma Aβ or pTau-181 and a measure of brain atrophy typical of AD.^36^ Thus, our findings suggest that amyloid may not be the predominant neuropathological component underlying cognitive decline among older adults with type 2 diabetes and overweight or obesity. However, most of the cohort was younger than 65 years at baseline, which is before significant amyloid accumulation occurs in most of the population. Therefore, we cannot rule out that blood markers of brain amyloid burden could be associated with cognitive decline in a later period of the lifespan among persons with type 2 diabetes.

Despite the lack of association of Aβ and pTau-181 with the cognitive composite *z* score or adjudicated cognitive impairment, increasing levels of both NfL and GFAP were associated with worsening on the composite *z* score and increased odds of cognitive impairment. NfL is a nonspecific biomarker of large caliber axonal neurodegeneration.^37^ Cross-sectional studies of individuals with type 2 or type 1 diabetes have shown that higher blood NfL levels are associated with worse cognition, but longitudinal studies have been lacking.^36,38^ In the current study, we did not find associations of plasma NfL at baseline and cognition measured 8 to 12 years later. However, we did find that increasing levels of NfL between baseline and 8 to 12 years were associated with declining cognitive function and an increased odds of adjudicated cognitive impairment 8 to 12 years later. These findings suggest that a single measure of NfL may not be a prognostic marker of cognitive impairment among individuals with type 2 diabetes and overweight or obesity but that serial assessments with increasing NfL levels may be a biomarker of cognitive decline in this population.

Elevated plasma GFAP levels have been associated with multiple neuropathologic findings (eg, astrocytosis, neuroinflammation, neurofibrillary tangles^39^) and with cognitive decline (both related and not related to AD) across multiple studies.^40^ However, few studies have examined GFAP as a biomarker of cognitive decline among individuals with diabetes. A cross-sectional study of older adults with type 1 diabetes found that a higher plasma GFAP level was associated with worse immediate memory, but no associations were found with other cognitive domains and no differences by cognitive impairment status.^36^ In the current study, we also did not find an association between baseline GFAP level and global cognition assessed at 8 to 12 years, but we did find that increasing levels of GFAP were associated with worse cognition at 8 to 12 years. These results suggest that change in plasma GFAP may be a useful biomarker of cognitive change among older adults with type 2 diabetes and overweight or obesity. Moreover, associations appeared stronger than those between change in NfL level and cognition.

### Strengths and Limitations

There are multiple strengths to the study. Look AHEAD was a randomized clinical trial of patients with type 2 diabetes and overweight or obesity using rigorous methods. Participants have been closely followed up for nearly 20 years, providing deep phenotyping and well-characterized outcomes. However, limitations also warrant consideration. First, there was no baseline cognitive assessment because it was not an initial focus of the trial. Therefore, participants were not excluded based on the presence of cognitive impairment at baseline. Nonetheless, our rigorous screening procedures would have effectively excluded those with clear impairment. Second, randomization facilitated comparable demographic and health characteristics across study arms at baseline, and there is no reason to suspect that the 2 groups would have differed in cognitive performance at baseline either. In addition, there may be other age-related chronic diseases that influence plasma levels of biomarkers for which we did not account. Third, we recognize that plasma pTau-217 is a better diagnostic marker for elevated brain amyloid. However, data on pTau-217 was not assayed in our dataset. Fourth, our findings may only be generalizable to a high-risk subset of the population (ie, older adults with type 2 diabetes and overweight or obesity). However, this group represents a population increasingly at risk for cognitive impairment.

## Conclusions

In this longitudinal cohort of older adults with type 2 diabetes and overweight or obesity, increasing levels of plasma NfL and GFAP, but not Aβ_42/40_ or pTau-181, were associated with decreasing cognitive composite scores and increased odds of MCI or probable dementia. Increasing levels of plasma NfL and GFAP among older adults with diabetes and overweight or obesity may be indicative of worsening cognition.

## Notes

Supplement 1. **eTable 1.** Baseline Characteristics of Participants by Inclusion Status **eTable 2.** Baseline Characteristics of Participants by Cognitive Adjudication Status **eAppendix.** Clinical Sites, the Coordinating Center, and Central Resource Centers

Supplement 2.
Data Sharing Statement
