# Supplementary material for: Alzheimer Disease Blood Biomarkers and Cognition Among Individuals With Diabetes and Overweight or Obesity
Source: JAMA Netw Open. 2025 Feb 6;8(2):e2458149. doi: 10.1001/jamanetworkopen.2024.58149 (PMC11803481; doi:10.1001/jamanetworkopen.2024.58149)
Supplement: Supplement 1. — eTable 1. Baseline Characteristics of Participants by Inclusion Status eTable 2. Baseline Characteristics of Participants by Cognitive Adjudication Status eAppendix. Clinical Sites, the Coordinating Center, and Central Resource Centers [file jamanetwopen-e2458149-s001.pdf]

Supplemental Online Content

Mielke MM, Evans JK, Neiberg RH, et al. Alzheimer disease blood biomarkers and cognition among individuals with diabetes and overweight or obesity. *JAMA Netw Open*. Published online February 6, 2025. doi:10.1001/jamanetworkopen.2024.58149

eTable 1. Baseline Characteristics of Participants by Inclusion Status

eTable 2. Baseline Characteristics of Participants by Cognitive Adjudication Status

eAppendix. Clinical Sites, the Coordinating Center, and Central Resource Centers

This supplemental material has been provided by the authors to give readers additional information about their work.

© 2025 Mielke MM et al. *JAMA Netw Open*.

eTable 1. Baseline Characteristics of Participants by Inclusion Status

Baseline Characteristic	***Included (n= 758)***	***Excluded (n=4148)***	***P-value***
Randomized Group, No. (%)			0.62
Diabetes Support and Education	373 (49.2%)	2082 (50.2%)	
Age, mean ± SD, years	61.5 ± 6.1	58.4 ± 6.7	<.001
Gender, No. (%)			0.11
Women	424 (55.9%)	2450 (59.1%)	
Men	334 (44.1%)	1698 (40.9%)	
Race, No. (%)			0.92
African American / Black (not Hispanic)	126 (16.6%)	678 (16.3%)	
American Indian / Native American / Alaskan Native	4 (0.5%)	20 (0.5%)	
Asian/Pacific Islander	6 (0.8%)	43 (1.0%)	
Hispanic	97 (12.8%)	579 (14.0%)	
White	511 (67.4%)	2741 (66.1%)	
Mixed	14 (1.8%)	87 (2.1%)	
Years of Education, No. (%)			<.001
< 13 years	187/735 (25.4%)	739/4068 (18.2%)	
13 - 16 years	263/735 (35.8%)	1540/4068 (37.9%)	
> 16 years	285/735 (38.8%)	1789/4068 (44.0%)	
Body Mass Index, mean ± SD, kg/m²	34.8 ± 5.3	36.1 ± 5.9	<.001
Systolic Blood Pressure, mean ± SD, mmHg	129.0 ± 17.7	129.1 ± 17.0	0.85
Diastolic Blood Pressure, mean ± SD, mmHg	69.5 ± 9.6	70.4 ± 9.5	0.02
HbA1c %, mean ± SD	7.2 ± 1.1	7.3 ± 1.2	0.06
Diabetes Duration 5+ years, No. (%)	427/752 (56.8%)	2203/4124 (53.4%)	0.09
Diabetes Treatment, No. (%)			0.91
No Meds	101/752 (13.4%)	540/4107 (13.1%)	
Oral Meds, No insulin	539/752 (71.7%)	2931/4107 (71.4%)	
Oral Meds + Insulin	112/752 (14.9%)	636/4107 (15.5%)	
Dyslipidemia, No. (%)	684 (90.2%)	3661 (88.3%)	0.12
Hypertension, No. (%)	637 (84.0%)	3465 (83.5%)	0.73
CVD History, No. (%)	127 (16.8%)	562 (13.5%)	0.02

© 2025 Mielke MM et al. *JAMA Netw Open*.

Baseline Characteristic	***Included (n= 758)***	***Excluded (n=4148)***	***P-value***
Peripheral Neuropathy, No. (%)	122 (16.1%)	736 (17.7%)	0.27
APOE ε4 carrier status, No. (%)	147/648 (22.7%)	779/3357 (23.2%)	0.77
eGFR <90, No. (%)	385/701 (54.9%)	2452/4136 (59.3%)	0.03

Abbreviations: *APOE* ε4, Apolipoprotein E gene, ε4 carrier status; CVD, cardiovascular disease; eGFR, estimated glomerular filtration rate; HbA1c, hemoglobin A1c; kg/m², kilogram per square meter; SD, standard deviation

© 2025 Mielke MM et al. *JAMA Netw Open*.

eTable 2. Baseline Characteristics of Participants by Cognitive Adjudication Status

Baseline Characteristic	***Total (n= 745)***	***Normal Cognitive Status (n= 448)***	***MCI or Probable Dementia (n= 297)***	***P-value***
Age, mean ± SD, years	61.5 ± 6.2	60.7 ± 5.9	62.7 ± 6.3	**<0.001**
Gender, No. (%)				**<0.001**
Women	413 (55.4%)	280 (62.5%)	133 (44.8%)	
Men	332 (44.6%)	168 (37.5%)	164 (55.2%)	
Race, No. (%)				0.46
African American / Black (not Hispanic)	125 (16.8%)	77 (17.2%)	48 (16.2%)	
American Indian / Native American / Alaskan Native	4 (0.5%)	3 (0.7%)	1 (0.3%)	
Asian/Pacific Islander	6 (0.8%)	3 (0.7%)	3 (1.0%)	
Hispanic	94 (12.6%)	63 (14.1%)	31 (10.4%)	
White	502 (67.4%)	296 (66.1%)	206 (69.4%)	
Mixed	14 (1.9%)	6 (1.3%)	8 (2.7%)	
Years of Education, No. (%)				**0.02**
< 13 years	181/722 (25.1%)	116/436 (26.6%)	65/286 (22.7%)	
13 - 16 years	257/722 (35.6%)	138/436 (31.7%)	119/286 (41.6%)	
> 16 years	284/722 (39.3%)	182/436 (41.7%)	102/286 (35.7%)	
Body Mass Index, mean ± SD, kg/m²	34.9 ± 5.3	34.6 ± 5.2	35.2 ± 5.5	0.15
HbA1c %, mean ± SD	7.2 ± 1.1	7.2 ± 1.1	7.2 ± 1.1	0.96
Diabetes Duration 5+ years, No. (%)	419/739 (56.7%)	245/442 (55.4%)	174/297 (58.6%)	0.40
Diabetes Treatment, No. (%)				0.19
No Meds	100/739 (13.5%)	63/443 (14.2%)	37/296 (12.5%)	
Oral Meds, No insulin	528/739 (71.4%)	322/443 (72.7%)	206/296 (69.6%)	
Oral Meds + Insulin	111/739 (15.0%)	58/443 (13.1%)	53/296 (17.9%)	
Dyslipidemia, No. (%)	671 (90.1%)	400 (89.3%)	271 (91.2%)	0.38
Hypertension, No. (%)	627 (84.2%)	369 (82.4%)	258 (86.9%)	0.10
Systolic Blood Pressure, mean ± SD, mmHg	129.1 ± 17.7	127.9 ± 16.7	131.0 ± 19.1	**0.02**
Diastolic Blood Pressure, mean ± SD, mmHg	69.6 ± 9.6	68.8 ± 9.7	70.7 ± 9.3	**<0.01**

© 2025 Mielke MM et al. *JAMA Netw Open*.

Baseline Characteristic	***Total (n= 745)***	***Normal Cognitive Status (n= 448)***	***MCI or Probable Dementia (n= 297)***	***P-value***
CVD History, No. (%)	126 (16.9%)	54 (12.1%)	72 (24.2%)	**<0.001**
Peripheral Neuropathy, No. (%)	120 (16.1%)	70 (15.6%)	50 (16.8%)	0.66
eGFR <90, mean ± SD, mg/DL	89.1 ± 14.6	90.0 ± 14.4	87.6 ± 14.9	0.45
APOE ε4 carrier status, No. (%)	145/638 (22.7%)	74/386 (19.2%)	71/252 (28.2%)	**<0.01**
$A\beta_{40}$, mean ± SD, pg/dl	66.7 ± 19.4	65.4 ± 18.2	68.5 ± 21.0	**0.04**
$A\beta_{42}$, mean ± SD, pg/dl	4.9 ± 1.4	4.8 ± 1.4	4.9 ± 1.4	0.63
$A\beta_{42/40}$, mean ± SD, pg/dl	0.1 ± 0.0	0.1 ± 0.0	0.1 ± 0.0	**0.03**
pTau-181, mean ± SD, pg/dl	9.3 ± 5.1	9.4 ± 5.0	9.1 ± 5.2	0.48
GFAP, mean ± SD, pg/dl	93.5 ± 40.5	90.3 ± 38.7	98.3 ± 42.6	**0.01**
NfL, mean ± SD, pg/dl	13.8 ± 6.5	13.3 ± 6.4	14.5 ± 6.5	**0.01**

Abbreviations: Aβ, Amyloid beta; *APOE* ε4, Apolipoprotein E gene, ε4 carrier status; CVD, cardiovascular disease; GFAP, glial fibrillary acidic protein; eGFR, estimated glomerular filtration rate; HbA1c, hemoglobin A1c; kg/m², kilogram per square meter; MCI, mild cognitive impairment; NfL, neurofilament light chain; pg/dl, picograms per deciliter; SD, standard deviation

© 2025 Mielke MM et al. *JAMA Netw Open*.

The clinical sites, the coordinating center, central resource centers, and federal sponsors are described below.

<u>Clinical Sites</u>

The Johns Hopkins Medical Institutions
Frederick L. Brancati, MD, MHS1*; Lee Swartz2; Lawrence Cheskin, MD3; Jeanne M. Clark, MD, MPH3; Kerry Stewart, EdD3; Richard Rubin, PhD3*; Jean Arceci, RN; Suzanne Ball; Jeanne Charleston, RN; Danielle Diggins; Mia Johnson; Joyce Lambert; Kathy Michalski, RD; Dawn Jiggetts; Chanchai Sapun.

Pennington Biomedical Research Center
George A. Bray, MD1; Allison Strate, RN2; Frank L. Greenway, MD3; Donna H. Ryan, MD3; Donald Williamson, PhD3; Timothy Church, MD3 ; Catherine Champagne, PhD, RD; Valerie Myers, PhD; Jennifer Arceneaux, RN; Kristi Rau; Michelle Begnaud, LDN, RD, CDE; Barbara Cerniauskas, LDN, RD, CDE; Crystal Duncan, LPN; Helen Guay, LDN, LPC, RD; Carolyn Johnson, LPN, Lisa Jones; Kim Landry; Missy Lingle; Jennifer Perault; Cindy Puckett; Marisa Smith; Lauren Cox; Monica Lockett, LPN

The University of Alabama at Birmingham
Cora E. Lewis, MD, MSPH1; Sheikilya Thomas MPH2; Monika Safford, MD3; Stephen Glasser, MD3; Vicki DiLillo, PhD3; Charlotte Bragg, MS, RD, LD; Amy Dobelstein; Sara Hannum, MA; Anne Hubbell, MS; Jane King, MLT; DeLavallade Lee; Andre Morgan; L. Christie Oden; Janet Raines, MS; Cathy Roche, RN, BSN; Jackie Roche; Janet Turman

Harvard Center
<u>Massachusetts General Hospital</u>
David M. Nathan, MD1; Enrico Cagliero, MD3; Kathryn Hayward, MD3; Heather Turgeon, RN, BS, CDE2; Linda Delahanty, MS, RD3; Ellen Anderson, MS, RD3; Laurie Bissett, MS, RD; Valerie Goldman, MS, RD; Virginia Harlan, MSW; Theresa Michel, DPT, DSc, CCS; Mary Larkin, RN; Christine Stevens, RN; Kylee Miller, BA; Jimmy Chen, BA; Karen Blumenthal, BA; Gail Winning, BA; Rita Tsay, RD; Helen Cyr, RD; Maria Pinto
<u>Joslin Diabetes Center</u>
Edward S. Horton, MD1; Sharon D. Jackson, MS, RD, CDE2; Osama Hamdy, MD, PhD3; A. Enrique Caballero, MD3; Sarah Bain, BS; Elizabeth Bovaird, BSN, RN; Barbara Fargnoli, MS,RD; Jeanne Spellman, BS, RD; Kari Galuski, RN; Ann Goebel-Fabbri, PhD; Lori Lambert, MS, RD; Sarah Ledbury, MEd, RD; Maureen Malloy, BS; Kerry Ovalle, MS, RCEP, CDE
<u>Beth Israel Deaconess Medical Center</u>
George Blackburn, MD, PhD1*; Christos Mantzoros, MD, DSc3; Ann McNamara, RN; Kristina Spellman, RD

University of Colorado Anschutz Medical Campus
James O. Hill, PhD1; Holly Wyatt, MD3; Marsha Miller, MS RD2; Brent Van Dorsten, PhD3; Judith Regensteiner, PhD3; Debbie Bochert; Ligia Coelho, BS; Paulette Cohrs,

© 2025 Mielke MM et al. *JAMA Netw Open*.

RN, BSN; Susan Green; April Hamilton, BS, CCRC; Jere Hamilton, BA; Eugene Leshchinskiy; Lindsey Munkwitz, BS; Loretta Rome, TRS; Terra Thompson, BA; Kirstie Craul, RD, CDE; Sheila Smith, BS; Cecilia Wang, MD

Baylor College of Medicine
John P. Foreyt, PhD1; Rebecca S. Reeves, DrPH, RD2; Molly Gee, MEd, RD2; Henry Pownall, PhD3; Ashok Balasubramanyam, MBBS3; Chu-Huang Chen, MD, PhD3; Peter Jones, MD3; Michele Burrington, RD, RN; Allyson Clark Gardner,MS, RD; Sharon Griggs; Michelle Hamilton; Veronica Holley; Sarah Lee; Sarah Lane Liscum, RN, MPH; Susan Cantu-Lumbreras; Julieta Palencia, RN; Jennifer Schmidt; Jayne Thomas, RD; Carolyn White

The University of Tennessee Health Science Center
University of Tennessee East
Karen C. Johnson, MD, MPH1; Carolyn Gresham, RN2; Mace Coday, PhD; Lisa Jones, RN; Lynne Lichtermann, RN, BSN; J. Lee Taylor, MEd, MBA
University of Tennessee Downtown
Abbas E. Kitabchi, PhD, MD1*; Helmut Steinberg, MD1;Ebenezer Nyenwe, MD3; Helen Lambeth, RN, BSN2; Moana Mosby, RN; Amy Brewer, MS, RD, LDN; Debra Clark, LPN; Andrea Crisler, MT; Debra Force, MS, RD, LDN; Donna Green, RN; Robert Kores, PhD; Renate Rosenthal, Ph.D.

University of Minnesota
Robert W. Jeffery, PhD1; Tricia Skarphol, MA2; John P. Bantle, MD3; J. Bruce Redmon, MD3; Richard S. Crow, MD3; Cindy Bjerk, MS, RD; Kerrin Brelje, MPH, RD; Carolyne Campbell; Melanie Jaeb, MPH, RD; Philip Lacher, BBA; Patti Laqua, RD; Therese Ockenden, RN; Birgitta I. Rice, MS, RPh, CHES; Carolyn Thorson, CCRP; Ann D. Tucker, BA; Mary Susan Voeller, BA

St. Luke's Roosevelt Hospital Center
Xavier Pi-Sunyer, MD1; Jennifer Patricio, MS2; Carmen Pal, MD3; Lynn Allen, MD;Janet Crane, MA, RD, CDN; Lolline Chong, BS, RD; Diane Hirsch, RNC, MS, CDE; Mary Anne Holowaty, MS, CN; Michelle Horowitz, MS, RD.

University of Pennsylvania
Thomas A. Wadden, PhD1; Barbara J Maschak-Carey, MSN, CDE 2; Robert I. Berkowitz, MD3; Gary Foster, PhD 3; Henry Glick, PhD 3; Shiriki Kumanyika, PhD, RD, MPH3; Brooke Bailer, PhD; Yuliis Bell; Chanelle Bishop-Gilyard, Psy.D; Raymond Carvajal, Psy.D; Helen Chomentowski; Renee Davenport; Lucy Faulconbridge, PhD; Louise Hesson, MSN, CRNP; Robert Kuehnel, PhD; Sharon Leonard, RD; Caroline Moran, BA; Monica Mullen, RD, MPH; Victoria Webb, BA.; Marion Vetter, MD, RD

University of Pittsburgh
John M. Jakicic, PhD1, David E. Kelley, MD1; Jacqueline Wesche-Thobaben, RN, BSN, CDE2; Lewis H. Kuller, MD, DrPH3; Andrea Kriska, PhD3; Amy D. Rickman, PhD, RD, LDN3, Lin Ewing, PhD, RN3, Mary Korytkowski, MD3, Daniel Edmundowicz, MD3;

© 2025 Mielke MM et al. *JAMA Netw Open*.

Rebecca Danchenko, BS; Tammy DeBruce; Barbara Elnyczky; David O. Garcia, MS; Patricia H. Harper, MS, RD, LDN; Susan Harrier, BS; Dianne Heidingsfelder, MS, RD, CDE, LDN; Diane Ives, MPH; Juliet Mancino, MS, RD, CDE, LDN; Lisa Martich, MS, RD; Tracey Y. Murray, BS; Karen Quirin; Joan R. Ritchea; Susan Copelli, BS, CTR

The Miriam Hospital/Alpert Medical School of Brown University
Rena R. Wing, PhD1; Caitlin Egan, MS2; Vincent Pera, MD3; Jeanne McCaffery, PhD3; Jessica Unick, PhD3; Ana Almeida; Kirsten Annis, BA; Barbara Bancroft, RN; April Bernier, BS; Sara Cournoyer, BA; Lisa Cronkite, BS; Jose DaCruz; Michelle Fisher, RN, CDOE; Linda Gay, MS, RD, CDE; Stephen Godbout, BS, BSN; Jacki Hecht, RN, MSN; Marie Kearns, MA; Deborah Maier-Fredey, MS, RD; Heather Niemeier, PhD; Suzanne Phelan, PhD; Angela Marinilli-Pinto, PhD; Deborah Ranslow-Robles; Hollie Raynor, PhD; Erica Robichaud, MSW, RD; Jane Tavares, BA; Kristen Whitehead

The University of Texas Health Science Center at San Antonio
Steven M. Haffner, MD1; Helen P. Hazuda, PhD1; Maria G. Montez, RN, MSHP, CDE2; Carlos Lorenzo, MD3; Charles F. Coleman, MS, RD; Domingo Granado, RN; Kathy Hathaway, MS, RD; Juan Carlos Isaac, RC, BSN; Nora Ramirez, RN, BSN; Ronda Saenz, MS, RD

VA Puget Sound Health Care System / University of Washington
Steven E. Kahn MB, ChB1; Brenda Montgomery, RN, MS, CDE2; Robert Knopp, MD3; Edward Lipkin, MD, PhD3; Dace Trence, MD3; Elaine Tsai, MD3; Valerie Baldisserotto, RD; Linda Castine, RN, BSN, CDE; Basma Fattaleh, BA; Kathy Fitzpatrick, RN; Diane Greenberg, PhD; Sukwan Nhan Jolley, RD; Hailey Mack, RD, MS, CDE; Ivy Morgan-Taggart; Anne Murillo, BS; Gretchen Otto, BS; Betty Ann Richmond, MEd; Jolanta Socha, BS; April Thomas, MPH, RD; Alan Wesley, BA; Diane Wheeler, RD, CDE

University of Southern California
Anne Peters, MD[1]; Valerie Ruelas, MSW, LCSW[2]; Siran Ghazarian Sengardi, MD[2]; Kathryn (Mandy) Graves Hillstrom, EdD, RD, CDE; Kati Konersman, MA, RD, CDE; Sara Serafin-Dokhan

Coordinating Center

Wake Forest University
Mark A. Espeland, PhD1; Judy L. Bahnson, BA, CCRP3; Lynne E. Wagenknecht, DrPH3; David Reboussin, PhD3; W. Jack Rejeski, PhD3; Alain G. Bertoni, MD, MPH3; Wei Lang, PhD3; Michael S. Lawlor, PhD3; David Lefkowitz, MD3*; Gary D. Miller, PhD3; Patrick S. Reynolds, MD3; Paul M. Ribisl, PhD3; Mara Vitolins, DrPH3; Daniel Beavers, PhD3; Haiying Chen, PhD, MM3; Delia S. West, PhD3; Lawrence M. Friedman, MD3; Ron Prineas, MD3; Tandaw Samdarshi, MD3; Kathleen M. Hayden, PhD3; Kathy M. Dotson, BA2; Amelia Hodges, BS, CCRP2; Dominique Limprevil-Divers, MA, MEd2; Karen Wall2; Carrie C. Williams, MA, CCRP2; Andrea Anderson, MS; Jerry M. Barnes, MA; Mary Barr; Tara D. Beckner; Cralen Davis, MS; Thania Del Valle-Fagan, MD; Melanie Franks, BBA; Candace Goode; Jason Griffin, BS; Lea Harvin,

© 2025 Mielke MM et al. *JAMA Netw Open*.

BS; Mary A. Hontz, BA; Sarah A. Gaussoin, MS; Don G. Hire, BS; Patricia Hogan, MS; Mark King, BS; Kathy Lane, BS; Rebecca H. Neiberg, MS; Julia T. Rushing, MS; Valery S. Effoe, MD, MS; Michael P. Walkup, MS; Terri Windham

Central Resources Centers

Central Laboratory, Northwest Lipid Metabolism and Diabetes Research Laboratories
Santica M. Marcovina, PhD, ScD1; Jessica Chmielewski2; Vinod Gaur, PhD4

Federal Sponsors

National Institute of Diabetes and Digestive and Kidney Diseases
Mary Evans, PhD; Barbara Harrison, MS; Van S. Hubbard, MD, PhD; Susan Z. Yanovski, MD

National Heart, Lung, and Blood Institute
Lawton S. Cooper, MD, MPH; Peter Kaufman, PhD, FABMR

Centers for Disease Control and Prevention
Edward W. Gregg, PhD; Ping Zhang, PhD

---

1 Principal Investigator
2 Program Coordinator
3 Co-Investigator
All other Look AHEAD staffs are listed alphabetically by site.
*Deceased
