# Supplementary material for: Alzheimer Disease Blood Biomarkers and Cognition Among Individuals With Diabetes and Overweight or Obesity
Source: JAMA Netw Open. 2025 Feb 6;8(2):e2458149. doi: 10.1001/jamanetworkopen.2024.58149 (PMC11803481; doi:10.1001/jamanetworkopen.2024.58149)
Supplement: Supplement 2. — Data Sharing Statement [file jamanetwopen-e2458149-s002.pdf]

Data Sharing Statement

Mielke. Alzheimer Disease Blood Biomarkers and Cognition Among Individuals With Diabetes and Overweight or Obesity. *JAMA Netw Open*. Published February 06, 2025. doi:10.1001/jamanetworkopen.2024.58149

Data
Data available: Yes
Data types: Deidentified participant data
How to access data: Core data from the Look AHEAD trial and its documentation are available at the NIDDK Data Repository (https://repository.niddk.nih.gov/home/). The Biomarker data will be posted there within one year of the publication of this manuscript.
When available: With publication

Supporting Documents
Document types: None

Additional Information
Who can access the data: researchers whose proposed use of the data has been approved.
Types of analyses: for a specified purpose
Mechanisms of data availability: after approval of a proposal and with a signed data access agreement.
